# Occupational Allergic Diseases in Kitchen and Health Care Workers: An Underestimated Health Issue

**DOI:** 10.1155/2013/285420

**Published:** 2013-11-11

**Authors:** Ugur Bilge, Ilhami Unluoglu, Nazan Son, Ahmet Keskin, Yasemin Korkut, Murat Unalacak

**Affiliations:** ^1^Department of Family Medicine, Faculty of Medicine, Eskisehir Osmangazi University, 9026480 Eskisehir, Turkey; ^2^Ankara Cankaya 4th Primary Care, 9006100 Ankara, Turkey; ^3^Department of Family Medicine, Faculty of Medicine, Dumlupinar University, 9043100 Kutahya, Turkey

## Abstract

*Objective*. This study evaluated the frequencies of allergic symptoms and rate of upper respiratory infections during the past year in the general population, kitchen workers (KW) and health care workers (HCW). *Methods*. The European Community Respiratory Health Survey (ECRHS) was used to inquire retrospectively about asthma and asthma-like symptoms and the number of treatments required for previous upper respiratory tract infections (URTI: acute pharyngitis, acute sinusitis, etc.) during the past year for health care workers, kitchen workers, and members of the general population. Adjusted odds ratios by gender, age, and smoking status were calculated. *Results*. 579 subjects (186 from the general population, 205 KW, and 188 HCW; 263 females, 316 males) participated in the study. Noninfectious (allergic) rhinitis was significantly higher in the HCW and KW groups than in the general population (*P* < 0.001). Cumulative asthma was significantly higher only in the HCW group (*P* < 0.05). In addition, the HCW and KW groups had significantly higher risks of ≥2/year URTI (OR: 1.59, 95% CI: 1.07–2.38 versus OR: 1.57, 95% CI: 1.05–2.38) than the general population. *Conclusion*. Occupational allergic respiratory diseases are an important and growing health issue. Health care providers should become familiar with workplace environments and environmental causes of occupational rhinitis and asthma.

## 1. Introduction

Allergic diseases are common and important health problems, as their prevalence is increasing worldwide. Allergic diseases also constitute an economic burden, as they affect quality of life and work life [1, 2].

Two major factors that can trigger the development and severity of allergic disease are host factors and environmental factors [3]. Environmental stimulants, including indoor allergens, can affect the development of allergic responses [3]. Indoor allergens involved in this process can include arthropod allergens, mammalian allergens, fungal allergens, and also occupational allergens [3–6]. Occupational asthma and allergic rhinitis are common respiratory diseases in industrialized countries [7]. Occupational allergic diseases can affect workers' productivity and quality of life [8]. Many occupations are at risk of allergic diseases such as occupational asthma and allergic rhinitis. These occupational groups include health care staff, kitchen workers, spray painters, bakers, laboratory technicians, and hairdressers [9, 10]. About 250 agents have been identified as causes of occupational allergic diseases [11]. 

According to studies on kitchen workers, there has been an increase in respiratory diseases like asthma, allergic rhinitiss and emphysema that may be associated with exposure to cooking fumes, disinfectants, cockroaches, and mice [3, 5]. During food preparation under high temperatures, harmful products like fat aerosols and aldehydes can be formed, which can cause respiratory symptoms [5]. 

Data on health care workers shows that an increased risk of allergic diseases and occupational allergy has become an important health problem [12]. Potential allergens for this group are latex, disinfectants, sterilants, pharmaceuticals, sensitizing metals, methacrylates, irritant aerosolized medications, and cleaning products [13–15]. Although occupational allergic diseases affect productivity and workers' health, their effects are usually underestimated.

In this study, we aimed to evaluate the frequencies of allergic symptoms, cigarette smoking, and upper respiratory tract infections (URTI) during the past year in the general population, kitchen workers, and health care workers.

## 2. Materials and Methods

This was an observational, cross-sectional study. A total of 579 subjects over the age of 18 participated. The subjects were recruited from kitchen workers (KW), health care workers (HCW) (doctors, nurses, and paramedics), and the general population. All data were collected in Eskisehir and Kutahya, which are in the northwestern part of Central Anatolia in Turkey. The socioeconomic levels of these cities were judged as average for Turkey. The participants were informed about the purpose of the study and voluntarily took part. A of total 250 HCW were approached, but only 188 of them agreed to participate. The kitchen workers were selected from three university kitchens (two in Eskisehir, one in Kutahya), which have 205 workers in total. All KW agreed to participate. To evaluate occupational effects on allergic symptoms and URTI, subjects who had worked for at least one year in the same job were included in the study. Kitchen workers' data were collected from three different kitchens, and health workers' data were collected from three different hospitals. The general population data were collected in the centers of the two cities. These subjects were selected by a simple random sampling method. A two-stage questionnaire was administered to people who volunteered to participate. In the first stage of the questionnaire, sociodemographic characteristics and smoking behavior of the participant were recorded; the number of upper respiratory tract infections (acute pharyngitis, acute sinusitis, etc.) that required treatment during the previous year was reported retrospectively. In the second stage of the questionnaire, the European Community Respiratory Health Survey (ECRHS) was administered to inquire about asthma and asthma-like symptoms. A chi-square test was used to examine the extent of relationships through a univariate analysis of the data obtained from the questionnaire. A logistic regression model was used for the multivariate analyses. Three main groups were included in the study, and the study design can be represented by a 2 × 3 contingency table. A power analysis was performed on this table using a chi-square test. Alpha was set at 0.05 and beta at 0.20, so the power was equal to 0.80 and the sample size of 571 was calculated to be enough for the 80% level of power. The categorical variables for all three groups were determined, and then adjusted odds ratios by gender, age, and smoking status (OR) for 95% confidence intervals (95% CI) were calculated. The median number of URTIs per year was 2, and ORs were calculated for all groups as 0-1/year and ≥2/year. All statistical analyses were performed using SPSS 16.0 for Windows. 

## 3. Results

A total of 579 subjects (186 from the general population, 205 KW and 188 HCW; 263 females, 316 males) were included in the study. Mean age ± standard deviation was 36.17 ± 10.65 years. Mean age ± standard deviations of the groups were 39.59 ± 14.80 years for the general population, 35.90 ± 7.12 for the kitchen workers, and 33.07 ± 7.56 for the health care workers. When comparisons between the general population and groups were made, noninfectious (allergic) rhinitis was significantly higher in the HCW and KW groups (*P* < 0.001). Cumulative asthma was significantly higher only in the HCW group (*P* < 0.05). Results of the questionnaire according to groups are presented in Table 1. When adjusted odds ratios by gender, age, and smoking status were calculated, being a HCW or a KW produced a 1.97-fold risk (OR: 1.97, 95% confidence interval (CI): 1.28–3.04) and 1.931-fold risk (OR: 1.85, 95% CI: 1.26–2.95) for noninfectious rhinitis, respectively. We did not observe any other significant relationships between groups. We also evaluated the URTI risk in the three groups and found that in the HCW and KW groups there were significantly higher risks of ≥2/year URTI (OR: 1.59, 95% CI: 1.07–2.38 versus OR: 1.57, 95% CI: 1.05–2.38) in comparison with the general population. However when adjusted odds ratios were calculated, we found that in the HCW group there is a significantly higher risk of ≥2/year URTI (OR: 1.56, 95% CI: 1.01–2.40). Average working years for groups, with standard deviations, were found to be 10.70  ±7.51 years for HCW and 12.36 ± 7.07 years for KW. We calculated the median working years for both of the workers' groups and found that working in a kitchen more than 12 years was related to a 2.30-fold risk of asthma-like symptoms (37.4% versus 57.9%, OR: 2.30, 95% CI: 1.31–4.05), compared to workers who had worked equal or less than 12 years. Two hundred fifteen of the subjects who participated in the study (33.9%) were regular smokers. When we evaluated the relationship between allergic diseases and allergic symptoms, we found a 4.193-fold risk of asthma-like symptoms (OR: 4.193, 95% CI: 2.93–6.00) in smokers, which was significant (*P* < 0.05).

## 4. Discussion

As 15% of adult asthma is attributed to the workplace environment, occupational respiratory diseases, especially asthma and allergic rhinitis, can be prevented through appropriate protective strategies. Allergic respiratory diseases can be caused by many allergens: aldehydes, flour, isocyanates, latex, persulphate, salts, disinfectants, sterilants, antibiotics, and cooking fumes, mostly seen in kitchen workers, health care workers, hairdressers, and cleaners [16–18]. According to studies on KW, food particle inhalation, cockroaches, and mice can cause respiratory symptoms such as nasal symptoms (rhinorrhea, sneezing, nasal congestion), lower respiratory and symptoms (cough, wheeze) and, rarely, anaphylaxis. Rhinitis can occur before occupational asthma, and rhinoconjunctivitis is also related to this condition [19]. In our data, asthma-like symptoms were significantly higher in the subjects who had worked for more than 12 years in kitchens, which can be harbingers of occupational asthma. In addition, kitchen workers have higher risk factors for lung cancer and emphysema [20–22]. Interestingly, we found that the cumulative asthma rate was lower than it was in the general population. The reason for such a finding may be that the workers with allergic symptoms had to change their jobs due to their occupational allergic symptoms or they did not seek work in such jobs. A study with bakers found that allergic symptoms can be the reason for changing jobs [23].

Health care workers have an increased risk of occupational allergic diseases since they were medical students. They have increased risks of contact dermatitis, allergic rhinitis, sinusitis, and bronchial asthma [24, 25]. Such diseases are caused by occupational allergens such as drugs, pollens, disinfectants, and hand cleaning substances. In our findings, being a HCW or KW was associated with an increased odds ratio of having noninfectious rhinitis. Occupational rhinitis is a health problem that receives less attention than occupational asthma [26]. It is an inflammatory disease characterized by intermittent or persistent symptoms, and it can be caused by particular working environments or exacerbated by occupational exposure. 

We found that being a KW or a HCW was associated with increased odds of having URTI. Chemical solvents, latex, antibiotics, and cooking oils are the major contributors to respiratory symptoms, and they can cause both allergic symptoms and upper respiratory tract infections [12, 27]. Also well, the presence of allergic symptoms is considered to be a risk factor for upper respiratory tract infections [28, 29].

The economic importance of work-related allergic diseases is growing, as they cause an increase in airway diseases like asthma, disease-related productivity loss, and a decrease in quality of life. Further, the medications used for treatment can cause adverse effects [30–34]. Prevention of occupational allergic diseases should be important for all healthcare providers. Controlling exposure in the workplace, identification of susceptible workers, and secondary prevention are the main means of prevention [26]. Secondary prevention includes periodical investigations of occupational allergic symptoms, early referral of symptomatic workers, and identification of workers who have possible asthma as well as occupational allergic diseases [26]. 

## 5. Conclusions

Occupational allergic respiratory diseases are an important health issue and their importance is growing. Health care providers should become familiar with the workplace environment and environmental causes of occupational rhinitis and asthma. Although giving appropriate medications is important, all health care providers should also encourage changes in the working environment to protect the workers' health. Paying attention to the working environment can result in decreases in occupational asthma and in progressive respiratory deterioration [35].

## Figures and Tables

**Table 1:** Distributions of allergic symptoms according to groups.

European Community Respiratory Health Survey (ECRHS) questionnaire	Questionnaire diagnoses	General population (*n*/*N*) (%)	Kitchen workers (*n*/*N*) (%)	Health care workers (*n*/*N*) (%)
Saying “Yes” to Q1 and/or Q2 and/or Q3 and/or Q4	With asthma-like symptoms	(77/186) 41.4	100/205 48.8	70/188 37.2
Current asthma: saying “Yes” to Q5 and Q7	With current asthma	(11/186) 5.9	6/205 2.9	10/188 5.7
Cumulative asthma: saying “Yes” to Q6	With cumulative asthma	(11/186) 5.9	5/205 2.4	19/188 10.1*
Noninfectious (allergic) rhinitis: saying “Yes” to Q8	With noninfectious rhinitis	(70/186) 37.6	108/205** 52.7	107/188** 56.9
Pruritic dermatitis and/or eczema: saying “Yes” to Q9	With pruritic dermatitis and/or eczema	(51/186) 27.4	61/205 29.8	58/188 30.9

*P < 0.05, **P < 0.001.
